# Association of Estimated Glomerular Filtration Rate with Hemoglobin Level in Korean Adults: The 2010–2012 Korea National Health and Nutrition Examination Survey

**DOI:** 10.1371/journal.pone.0150029

**Published:** 2016-04-29

**Authors:** Sang Youb Han, Se Won Oh, Jae Won Hong, Seong Yoon Yi, Jung Hyun Noh, Hye Ran Lee, Dong-Jun Kim

**Affiliations:** Department of Internal Medicine, Inje University Ilsan-Paik Hospital, Goyang, Korea; The University of Tokyo, JAPAN

## Abstract

**Purpose:**

Little is known about anemia in patients with early renal dysfunction. We aimed to investigate the association of hemoglobin level and anemia prevalence with estimated glomerular filtration rate (eGFR) decline using a nation-wide representative sample of the adult Korean population.

**Methods:**

In total, 17,373 participants (7,296 men; weighted n = 18,330,187; mean age, 44.2±0.3 years; 9,886 women, weighted n = 18,317,454; mean age, 46.9±0.3 years) were included. eGFR was divided into 5 groups: Group 1, ≥105; Group 2, 90–104; 75–89; Group 4, 60–74; and Group 5, <60 mL/min/1.73m^2^.

**Results:**

The weighted anemia prevalence rates were 2.6% in men and 12.8% in women. In men, the weighted hemoglobin level increased with a decrease in eGFR; this value peaked at an eGFR of 60–89 mL/min/1.73m^2^ and decreased thereafter at an eGFR of <60 mL/min/1.73m^2^ (15.19±0.03, 15.35±0.03, 15.53±0.03, 15.52±0.06, and 14.90±0.12 g/dL from Groups 1 to 5) after adjustment for age, college graduation, cancer history, current smoking, waist circumference, serum cholesterol level, serum triglyceride level, and diastolic blood pressure. In women, the weighted hemoglobin level increased with a decrease in eGFR; this value peaked with an eGFR of 75–89 mL/min/1.73m^2^ and decreased thereafter (12.90±0.03, 13.08±0.02, 13.20±0.04, 13.14±0.05, and 12.47±0.11 g/dL from Groups 1 to 5) after adjustment for menstruation, pregnancy, estrogen replacement, and the above-mentioned variables. In both sexes, the weighted prevalence of anemia with an eGFR of 60–104 mL/min/1.73m^2^ was significantly lower than that with an eGFR of ≥105 mL/min/1.73m^2^ (men, 3.2±0.4%, 1.9±0.3%, 1.8±0.3%, 2.0±0.9%, and 18.1±3.1%; women, 14.0±0.8%, 11.2±0.7%, 10.5±1.0%, 13.2±1.6%, and 32.3±3.2% from Groups 1 to 5).

**Conclusions:**

We noted a compensatory increase in the hemoglobin level with a minor decline in kidney function (in the range of eGFR ≥60 mL/min/1.73m^2^) prior to a marked decrease in hemoglobin level with severe renal dysfunction.

## Introduction

Anemia is a common complication of chronic kidney disease (CKD), and is associated with an increased risk of cardiovascular disease (CVD) and mortality, particularly in high-risk populations [1,2]. The National Health and Nutrition Examinational Survey (NHANES) III in the USA reported that the prevalence of anemia is increased in subjects with a glomerular filtration rate (GFR) of <60 mL/min/1.73m^2^[3]. Anemia associated with decreased renal function is attributed to the reduced production of erythropoietin. However, as renal function deteriorates, anemia may be attributed to other factors such as iron deficiency, blood loss, and inflammation [4,5].

Although it is well known that the prevalence of anemia is higher among cases with an eGFR of <60 mL/min/1.73m^2^, information on this association during early renal dysfunction is scarce, and only few studies have reported on this issue. Furthermore, the findings of most studies are not applicable to the general population, and most studies did not consider the factors that may affect the hemoglobin level [6]. In the present study, we aimed to investigate the association of hemoglobin level and prevalence of anemia with the decline in eGFR using a nation-wide representative sample of the adult Korean population.

## Subjects and Methods

### Study population and data collection

This study is based on data from the 2010–2012 Korea National Health and Nutrition Examination Survey (KNHANES), which is a cross-sectional and nationally representative survey conducted by the Korean Center for Disease Control and Prevention. The following information is reproduced from our previous work [7]. The KNHANES has been conducted periodically since 1998 to assess the health and nutritional status of the civilian non-institutionalized population of Korea. A representative population was recruited using population-allocation-systematic sampling with multistage stratification. The study was approved by the Institutional Review Board of Ilsan-Paik Hospital (IB-1412-056). The KNHANES dataset was made available at the request of the investigator after approval. Since the dataset did not include any personal information and the participants in KNHANES had already consented, our study was exempted by the board from the necessity to obtain participant consent.

### Laboratory tests

Hemoglobin (Hb) was measured by XE-2100D (Sysmex, Kobe, Japan) in Neodin. The serum creatinine concentrations of a random sample were measured using a colourimetric method (Hitachi Automatic Analyser, Hitachi, Japan). Serum iron and Unsaturated iron binding capacity levels were measured by using the direct bathophenanthroline method (Hitachi Automatic Analyzer 7600, Hitachi, Japan), whereas serum ferritin levels were measured by using an immunoradiometric assay (1470 WIZARD gamma-counter, PerkinElmer, Finland).

The eGFR was determined using the Chronic Kidney Disease Epidemiology Collaboration formula: eGFR (ml/min/1.73 m^2^) = 141 × min(SCr/k,1)^a^ × max(SCr/k,1)^-1.209^ × 0.993^Age^ [1.018 if Female] × [1.159 if Black] (SCr: serum creatinine [mg/dL], k: 0.7 for females and 0.9 for males, a: -0.329 for females and -0.411 for males, min: minimum of SCr/k or 1, and max: maximum of SCr/k or 1) [8]. The results of the detailed health interviews, physical examinations, and other laboratory procedures have been published elsewhere [9].

Anemia was defined as a hemoglobin level of less than 13 g/dL in men and less than 12 g/dL in women according to the criteria of the World Health Organization [10]. Iron deficiency anemia (IDA) was defined as a transferrin saturation of less than 10% or ferritin levels of less than 15 ug/L. Obesity was defined as a BMI of ≥25 kg/m^2^[11]. The eGFR was classified into 5 groups: Group 1, ≥105; Group 2, 90–104; Group 3, 75–89; Group 4, 60–74; Group 5, <60 mL/min/1.73m^2^.

### Statistical analyses

The following information is reproduced from our previous work [7]. The KNHANES participants were not randomly sampled. The survey was designed using a complex, stratified, multistage probability sampling model; thus, individual participants were not equally representative of the Korean population. To obtain representative prevalence rates from the dataset, it was necessary to consider the power of each participant (sample weight) as representative of the Korean population. After approval was obtained from the Korea Centers for Disease Control and Prevention, we received a survey dataset that included information about the survey location; strata by age, sex, and various other factors; and the sample weight for each participant. The survey sample weights, calculated while considering the sampling rate, response rate, and age/sex proportions of the reference population (2005 Korean National Census Registry), were used in all analyses to provide representative estimates of the non-institutionalized Korean civilian population.

Statistical analyses were performed using SPSS software (ver. 21.0 for Windows; SPSS, Chicago, IL, USA). To compare the weighted prevalence of anemia according to age and sex, the chi-square test was performed. We compared age- and sex-adjusted clinical characteristics according to the presence of anemia for each sex using a general linear model (GLM). Weighted and adjusted hemoglobin level, weighted and adjusted prevalence of anemia, and weighted and adjusted prevalence of IDA and non-IDA were compared among the 5 eGFR groups using the GLM. All tests were two-sided, and *P*-values of < .05 were considered to indicate statistical significance.

## Results

### Demographics and clinical characteristics of the study population

A total of 25,534 people participated in the KNHANES V-2, 2010–2012. Of those, 19,599 participants were aged 19 years and older. Among them, a total of 17,373 participants (weighted number, 36,647,641) who completed the laboratory examination were finally included in the analysis. The overall mean age was 45.0±0.2 years (95% confidence interval [CI], 44.7–46.5). The weighted mean age of the men (unweighted n = 7,296/weighted n = 18,330,187) and women (unweighted n = 9,886/weighted n = 18,317,454) were 44.2±0.3 and 46.9±0.3 years, respectively. The demographics and clinical characteristics of the study population are presented in Table 1.

**Table 1:** Weighted clinical characteristics of Korean men and women.

	Men	Women	Total
*N*, (unweighted/weighted)	7,483/18,330,187	9,895/18,317,454	17,378/36,647,641
Age (years)	44.2±0.3	46.9±0.3	45.0±0.2
Heavy Alcohol drinking (%)	12.4±0.5	2.2 ±0.2	7.3±0.3
Regular exercise (%)	9.7±0.5	7.7±0.4	8.7±0.3
Current smoking (%)	45.1±0.7	7.0±0.4	26.1±0.5
College graduation (%)	35.1±0.8	28.3±0.7	31.7±0.7
Previous cancer history (%)	1.8±0.2	2.9±0.2	2.3±0.1
Pregnancy (%)	-	1.0±0.1	-
Lactation (%)	-	1.8±0.2	-
Menstruation (%)	-	56.6±0.7	-
Estrogen replacement (%)	-	6.8±0.3	-
Waist circumference (cm)	84.2±0.2	77.9±0.2	81.0±0.1
Body mass index (kg/m^2^)	24.1±0.1	23.3±0.1	23.7±0.1
Obesity (%)	36.2±0.7	27.7±0.6	31.9±0.5
Systolic BP (mmHg)	120.7±0.3	115.6±0.3	118.2±0.2
Diastolic BP (mmHg)	79.4±0.2	73.6±0.2	76.5±0.1
Anti-hypertensive drugs (%)	12.9±0.5	15.5±0.5	14.2±0.4
Hypertension (%)	30.1±0.7	23.1±0.6	26.6±0.5
Fasting plasma glucose (mg/dl)	98.7±0.3	94.9±0.3	96.8±0.2
Anti-diabetes drugs (%)	5.4±0.3	4.7±0.3	5.1±0.2
Diabetes (%)	10.5±0.4	8.3±0.3	9.4±0.3
Serum total cholesterol (mg/dl)	187.5±0.6	188.5±0.5	188.0±0.4
Serum Triglyceride (mg/dl)	155.5±1.9	112.2±1.2	133.9±1.2
Serum HDL-cholesterol (mg/dl)	49.4±0.2	55.6±0.2	52.5±0.1
Anti-lipid drugs (%)	3.6±0.2	5.2±0.3	4.4±0.2
Hb (g/dl)	15.4 ± 0.1	13.0±0.1	14.2±0.1

Data are expressed as means with SEM. Abbreviations: Heavy alcohol drinking: drinking four or more times per week, Regular exercise: exercising as five or more times per week, obesity: BMI ≥ 25 kg/m2 or more, hypertension: systolic blood pressure (BP) ≥140 mmHg, diastolic blood pressure ≥90 mmHg, or use of antihypertensive medications irrespective of blood pressure, Diabetes: fasting plasma glucose (FPG) ≥7.0 mmol/l, current anti-diabetes medication, or a previous diagnosis of diabetes by a doctor, HDL-C: high density lipoprotein-cholesterol.

### Prevalence of anemia according to age and sex

The weighted prevalence of anemia was 2.6% (95% CI, 2.2–3.1) in men and 12.8% (95% CI, 12.0–13.6) in women (Table 2). In men, the weighted prevalence of anemia significantly increased with increasing age (0.9% [95% CI, 0.5–1.6] in younger adults, 2.6% [95% CI, 1.9–3.4] in middle-aged adults, and 11.1% [95% CI, 9.4–13.1] in older adults). In women, the weighted prevalence of anemia was the lowest in middle-aged adults (14.1% [95% CI, 12.8–15.4] in younger adults, 9.7% [95% CI, 8.6–10.9] in middle-aged adults, and 15.7% [95% CI, 13.9–17.8] in older adults)

**Table 2:** Estimated prevalence of anemia in the Korean adults by age and sex.

		Men		Women		*P*	Total	
		Unweighted prevalence%	Weighted prevalence % [95% CI]	Unweighted prevalence %	Weighted prevalence % [95% CI]		Unweighted prevalence %	Weighted prevalence% [95% CI]
***n***		7,396	18,330,187	9,886	18,317,454		17,378	36,647,641
**Total (%)**		4.0	2.6 [2.2–3.1]	12.6	12.8 [12.0–13.6]	<0.001	8.9	7.7 [7.2–8.2]
**Age, years**	19–39 (%/*n*)	0.7 (20/2,940)	0.9 [0.5–1.6] (87,994/9,783,718)	14.9 (599/4,021)	14.1 [12.8–15.4] (1,281,683/9,117,711)	<0.001	8.9 (619/6,961)	7.3 [6.6–8.0] (1,369,677/18,856,429)
	40–64 (%/*n*)	2.6 (74/2,805)	2.6 [1.9–3.4] (168,227/6,559,145)	9.3 (342/3,659)	9.7 [8.6–10.9] (622,510/6,417,156)	<0.001	6.4 (416/6,464)	6.1 [5.5–6.8] (790,736/12,976,301)
	≥ 65 (%/*n*)	11.6 (202/1,738)	11.1 [9.4–13.1] (225,298/2,032,324)	13.7 (303/2,215)	15.7 [13.9–17.8] (438,080/2,782,587)	<0.001	12.8 (505/3,953)	13.8 [12.4–15.3] (663,378/4,814,911)

*P* of weighted prevalence between each gender

The weighted prevalence of IDA differed between the sexes. IDA was more common among women (men: 0.7% [95% CI, 0.5–0.9] vs. women: 8.0% [95% CI, 7.3–8.7]). In men, the weighted prevalence of IDA significantly increased with increasing age (0.4% [95% CI, 0.2–0.8] in younger adults, 0.8% [95% CI, 0.5–1.2] in middle-aged adults, and 1.9% [95% CI, 1.2–2.9] in older adults). However, in women, the prevalence of IDA decreased with increasing age (10.9% [95% CI, 9.8–12.1] in younger adults, 6.2% [95% CI, 5.3–7.3] in middle-aged adults, and 2.8 [95% CI, 2.0–3.7] in older adults).

### Demographic and clinical characteristics according to the presence of anemia

In weighted and age-adjusted comparisons, we noted that although anemic individuals were more likely to have a history of cancer, they were less likely to be heavy alcohol drinkers, current smokers, obese, and college graduates, and were also less likely to have lower diastolic blood pressure, serum total cholesterol levels, and serum triglyceride levels (Table 3).

**Table 3:** Weighted age and age-adjusted demographic and clinical characteristics of Korean men and women by the presence of anemia.

		Men			Women	
	No anemia	Anemia	*p*	No anemia	Anemia	*p*
*N*, (unweighted/weighted)	7,187/17,848,668	296/481,519		8,651/15,975,182	1,244/2,342,272	
Age (years)	43.7± 0.3	60.9± 1.8	<0.001	45.8 ± 0.3	46.2± 0.6	0.594
Heavy alcohol drinking (%)	12.6±0.5	4.7±2.8	0.006	2.4±0.2	0.9±0.3	<0.001
Regular exercise (%)	9.7±0.5	9.9±2.7	0.933	7.6±0.4	8.5±1.1	0.400
Current smoking (%)	45.4±0.7	37.7±3.3	0.021	7.5±0.4	3.9±0.7	<0.001
College graduation (%)	35.4±0.8	22.4±2.6	<0.001	27.8±0.7	31.7±1.6	0.011
Previous cancer history (%)	1.7±0.2	5.9±1.5	0.007	2.8±0.2	3.2±0.5	0.545
Pregnancy (%)	-	-		0.7±0.1	2.4±0.1	0.003
Lactation (%)	-	-		1.9±0.2	1.2±0.4	0.090
Menstruation (%)	-	-		55.3±0.5	65.6±1.3	<0.001
Estrogen replacement (%)	-	-		7.3±0.3	3.4±0.6	<0.001
Waist circumference (cm)	84.3±0.2	78.3±0.7	<0.001	78.1±0.2	76.3±0.4	<0.001
Body mass index (kg/m^2^)	24.2±0.1	22.0±0.2	<0.001	23.3±0.1	22.8±0.1	<0.001
Obesity (%)	36.7±0.7	14.3±2.6	<0.001	28.4±0.6	22.7±1.5	<0.001
Systolic BP (mmHg)	120.8±0.2	118.9±1.5	0.219	115.8±0.2	114.5±0.5	0.020
Diastolic BP (mmHg)	79.6±0.2	72.9±0.7	<0.001	74.1±0.2	70.9±0.4	<0.001
Anti-hypertensive drugs (%)	12.9±0.4	14.6±3.0	0.573	15.6±0.4	15.5±0.9	0.951
Hypertension (%)	30.0±0.7	26.0±3.7	0.280	23.0±0.5	21.0±1.1	0.054
Fasting plasma glucose (mg/dl)	98.8±0.3	95.2±1.9	0.059	94.9±0.3	94.4±0.7	0.479
Anti-diabetes drugs (%)	5.2±0.3	12.6±2.7	0.008	4.5±0.3	6.6±0.8	0.011
Diabetes (%)	10.0±0.4	16.0±3.2	0.105	8.0±0.3	9.0±0.8	0.750
Serum total cholesterol (mg/dl)	188.3±0.6	156.4±3.1	<0.001	189.8±0.5	180.1±1.2	<0.001
Serum Triglyceride (mg/dl)	157.1±1.9	95.1±6.5	<0.001	114.0±1.3	100.4±2.4	<0.001
Serum HDL-cholesterol (mg/dl)	49.5±0.2	49.3±1.3	0.924	55.7±0.2	55.1±0.4	0.207
Anti-lipid drugs (%)	3.6±0.2	4.8±2.1	0.580	5.4±0.3	3.7±0.7	0.014
Hb (g/dl)	15.4±0.1	12.2±0.1	<0.001	13.3±0.1	10.9±0.1	<0.001

Data are expressed as means with SEM. Abbreviations: Heavy alcohol drinking: drinking four or more times per week, Regular exercise: exercising as five or more times per week, obesity: BMI ≥ 25 kg/m2 or more, hypertension: systolic blood pressure (BP) ≥140 mmHg, diastolic blood pressure ≥90 mmHg, or use of antihypertensive medications irrespective of blood pressure, Diabetes: fasting plasma glucose (FPG) ≥7.0 mmol/l, current anti-diabetes medication, or a previous diagnosis of diabetes by a doctor, HDL-C: high density lipoprotein-cholesterol.

Moreover, we found that anemic women were likely to have higher a rate of menstruation and pregnancy, and were more likely to be college graduates. However, they were less likely to be heavy alcohol drinkers, current smokers, and obese, and were also less likely to have lower systolic and diastolic BP, serum total cholesterol levels, and serum triglyceride levels.

### Hemoglobin level according to eGFR

**Fig 1:**
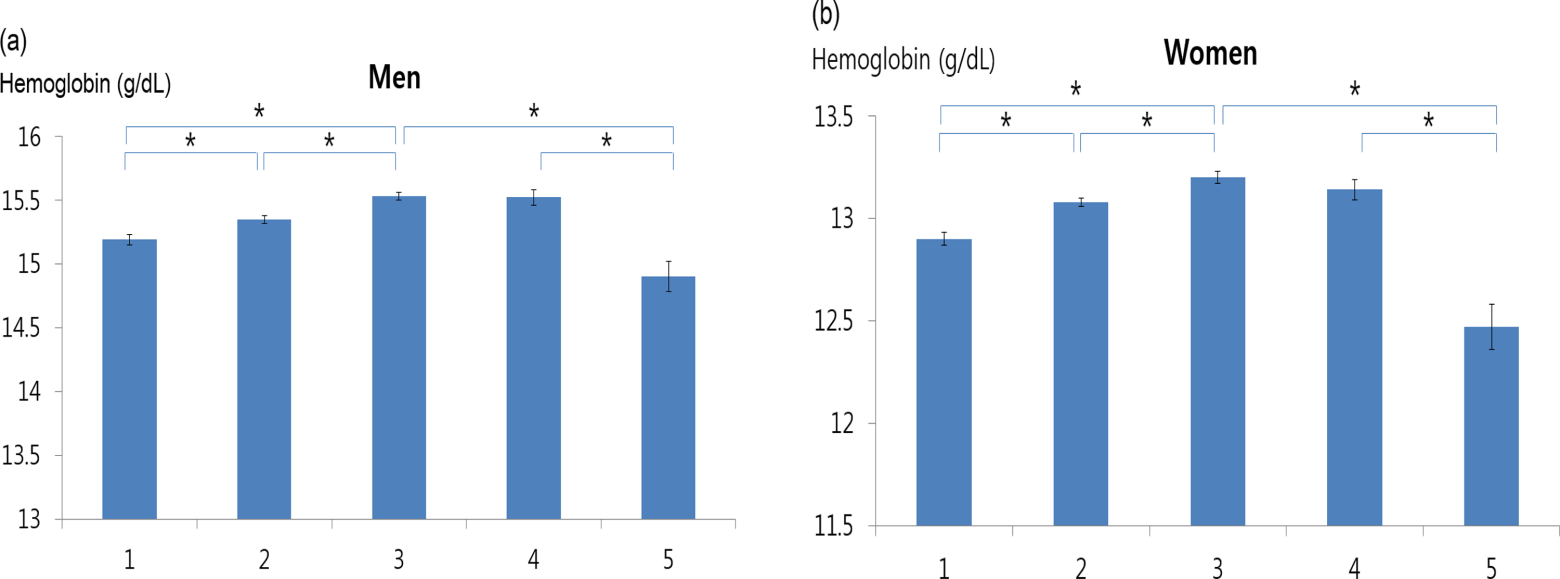
Adjusted hemoglobin levels according to estimated glomerular filtration rate (eGFR). The hemoglobin value was adjusted for age, college graduation, cancer history, current smoking, waist circumference, serum cholesterol level, serum triglyceride level, and diastolic blood pressure in men, and additionally for menstruation, pregnancy, and estrogen replacement in women. Group 1, eGFR of ≥105; Group 2, eGFR of 90–104; Group 3, eGFR of 75–89; Group 4, eGFR of 60–74; Group 5, eGFR of <59 mL/min/1.73m^2^. * P < .01, ** P < .05.

In men, the weighted hemoglobin level increased with a decrease in eGFR (at an eGFR of ≥90 mL/min/1.73m^2^); the weighted hemoglobin level then peaked at an eGFR of 60–89 mL/min/1.73m^2^, and decreased thereafter (at an eGFR of <60 mL/min/1.73m^2^) (15.19±0.03 in Group 1, 15.35±0.03 in Group 2, 15.53±0.03 in Group 3, 15.52±0.06 in Group 4, and 14.90±0.12 g/dl in Group 5) after adjustment for age, college graduation, cancer history, current smoking, waist circumference, serum cholesterol level, serum triglyceride level, and diastolic BP (Table 4, Fig 1).

**Table 4:** Weighted and adjusted Hemoglobin (g/dl) by eGFRin each sex.

eGFR	105-	90–104	75–89	60–74	-59	*P*
Men						
*N (unweighted/weighted)*	1,506/5,136,574	2,629/7,005,407	2,203/4,411,351	854/1,368,528	291/408,327	
Model 1	15.46 ± 0.04**	15.39 ± 0.03	15.37 ± 0.03	15.08±0.06*	14.08±0.12*,#	<0.001
Model 2	15.14 ± 0.04*,§	15.38 ± 0.03*	15.57 ± 0.03	15.50±0.06	14.74±0.13*,#	<0.001
Model 3	15.15 ± 0.04*,§	15.37 ± 0.03*	15.56 ± 0.03	15.50±0.06	14.77±0.13*,#	<0.001
Model 4	15.19 ± 0.03*,§	15.35 ± 0.03*	15.53 ± 0.03	15.52±0.06	14.90±0.12*,#	<0.001
Women						
*N (unweighted/weighted)*	3,243/7,365,548	3,409/6,093,879	2,180/3,325,542	766/1,037,700	297/405,079	
Model 1	12.86±0.03*,§	13.1 ±0.02	13.22±0.03	13.10±0.05**	12.26±0.11*,#	<0.001
Model 2	12.85±0.03*,§	13.15±0.02	13.22±0.04	13.12±0.06	12.28±0.12*,#	<0.001
Model 3	12.84±0.03*,§	13.15±0.02**	13.24±0.04	13.13±0.05	12.30±0.11*,#	<0.001
Model 4	12.87±0.03*,§	13.11±0.02*	13.23±0.04	13.16±0.05	12.45±0.11*,#	<0.001
Model 5	12.90±0.03*,§	13.08±0.02*	13.20±0.04	13.14±0.05	12.47±0.11*,#	<0.001

Model 1: unadjusted

Model 2: age-adjusted

Model 3: age, college graduation, previous cancer history, current smoking, and waist circumference

Model 4: serum total cholesterol, serum triglyceride, diastolic BP, and variables in model3

Model 5: menstruation, pregnancy, estrogen replacement, and variables in model 4

The hemoglobin level was the highest in the group of an eGFR≥105 mL/min/1.73m^2^ in men, and 75–89 mL/min/1.73m^2^ in women. After adjustment, the hemoglobin level was the highest in the group of an eGFR of 75–89 mL/min/1.73m^2^in both sex. P value means P for difference.

* P<0.01 vs. Group 3

** P<0.05 vs. Group 3

§ P<0.01 vs. Group 2

# P<0.01 vs. Group 4.

In women, the weighted hemoglobin level increased with a decrease in eGFR (at an eGFR of ≥90 mL/min/1.73m^2^); the weighted hemoglobin level then peaked at an eGFR of 75–89 mL/min/1.73m^2^ (Group 3), and decreased thereafter (at an eGFR of <75 mL/min/1.73m^2^) (12.90±0.03 in Group 1, 13.08±0.02 in Group 2, 13.20±0.04 in Group 3, 13.14±0.05 in Group 4, and 12.47±0.11 g/dl in Group 5) after adjustment for menstruation, pregnancy, estrogen replacement, and the above-mentioned variables (Table 4, Fig 1).

### Prevalence of anemia, IDA, and non-IDA according to eGFR

In both sexes, the weighted and adjusted prevalence of anemia at an eGFR of 60–104 mL/min/1.73m^2^ was significantly lower than that at an eGFR of ≥105 mL/min/1.73m^2^, whereas the weighted prevalence of anemia at an eGFR of <60 mL/min/1.73m^2^ was markedly higher than that at an eGFR of 60–104 mL/min/1.73m^2^ (men, 3.2±0.4% in Group 1, 1.9±0.3% in Group 2, 1.8±0.3% in Group 3, 2.0±0.9% in Group 4, and 18.1±3.1% in Group 5; women, 14.0±0.8% in Group 1, 11.2±0.7% in Group 2, 10.5±1.0% in Group 3, 13.2±1.6% in Group 4, and 32.3±3.2% in Group 5).

**Fig 2:**
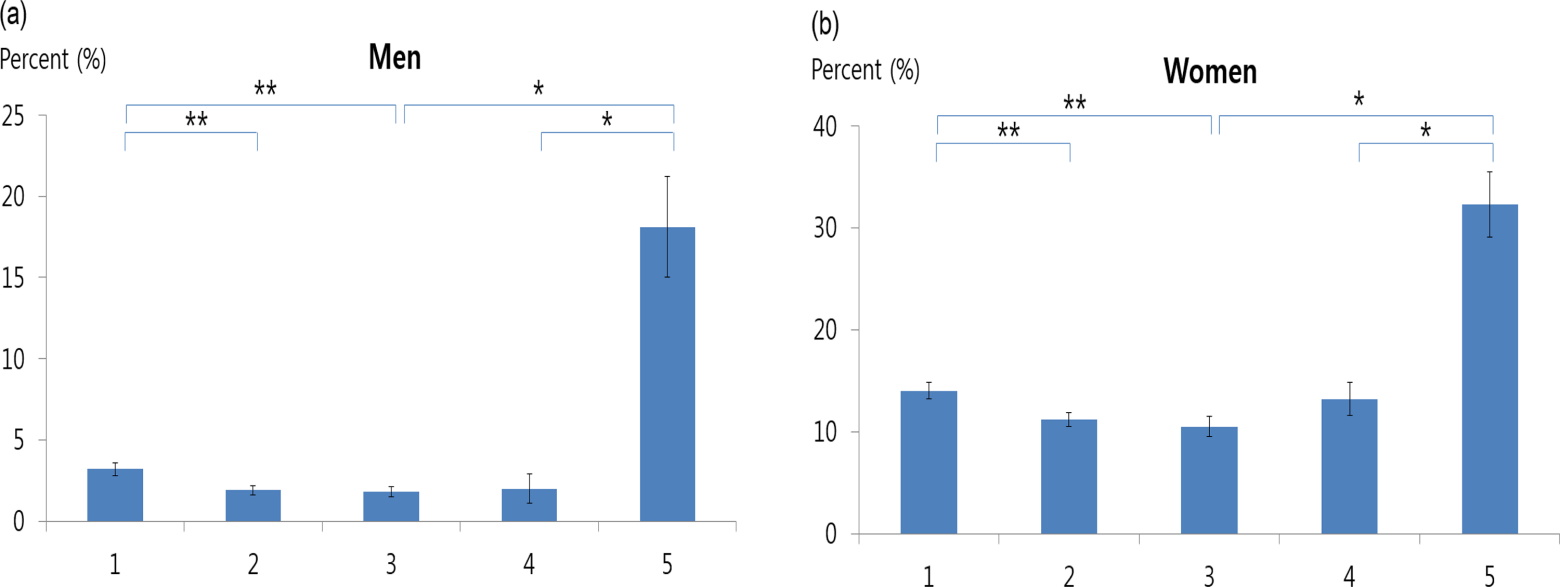
Weighted prevalence of anemia according to estimated glomerular filtration rate (eGFR). The weighted prevalence of anemia was adjusted for age, college graduation, cancer history, current smoking, waist circumference, serum cholesterol level, serum triglyceride level, and diastolic blood pressure in men, and additionally for menstruation, pregnancy, and estrogen replacement in women. Group 1, eGFR of ≥105; Group 2, eGFR of 90–104; Group 3, eGFR of 75–89; Group 4, eGFR of 60–74; Group 5, eGFR of <59 mL/min/1.73m^2^. * P < .01, ** P < .05.

The weighted prevalence of IDA did not differ according to the eGFR in men but was likely to decrease with a decline in eGFR in women (Table 5, Fig 2).

**Table 5:** Weighted and adjusted prevalence of anemia, iron deficiency anemia, and non-iron deficiency anemia in each sex.

		eGFR	105-	90–104	75–89	60–74	-59	*p*
**Men**	Model 1	Anemia	1.6±0.4**	1.6 ± 0.3**	2.8 ± 0.4	4.8 ± 0.9**	24.1± 3.2*#	<0.001
		Iron deficiency anemia	0.5±0.2	0.5 ± 0.2	0.8 ± 0.2	1.2 ± 0.5	3.9 ± 1.6	0.134
		Non-iron deficiency anemia	1.1±0.3**	1.1 ± 0.2**	2.1 ± 0.3	3.7 ± 0.7**	20.2 ± 2.9*#	<0.001
	Model 2	Anemia	3.7±0.5*§	1.7 ± 0.3	1.4 ± 0.3	2.0 ± 0.9	19.7 ± 3.3*#	<0.001
		Iron deficiency anemia	0.9±0.3	0.5 ± 0.2	0.5 ± 0.2	0.6 ± 0.5	3.0 ± 1.7	0.283
		Non-iron deficiency anemia	2.8±0.4*§	1.2 ± 0.2	1.0 ± 0.3	1.4 ± 0.7	16.7 ± 3.0*#	<0.001
	Model 3	Anemia	3.5±0.5*§	1.8 ± 0.3	1.6 ± 0.3	2.1 ± 0.9	18.7 ± 3.1*#	<0.001
		Iron deficiency anemia	0.9±0.2	0.5 ± 0.2	0.5 ± 0.2	0.7 ± 0.5	3.1 ± 1.7	0.379
		Non-iron deficiency anemia	2.7±0.4*§	1.2 ± 0.3	1.1 ± 0.3	1.4 ± 0.7	15.7 ± 2.8*#	<0.001
	Model 4	Anemia	3.2±0.4**§§	1.9 ± 0.3	1.8 ± 0.3	2.0 ±0.9	18.1 ± 3.1*#	<0.001
		Iron deficiency anemia	0.8±0.2	0.6 ± 0.2	0.6 ± 0.2	0.7 ± 0.5	2.9 ± 1.7	0.558
		Non-iron deficiency anemia	2.5±0.3*§	1.3 ± 0.3	1.3 ± 0.3	1.4 ± 0.7	15.2 ± 2.8*#	<0.001
**Women**	Model 1	Anemia	14.6±0.7*§	10.1 ± 0.6	10.4 ± 0.9	14.0 ± 1.5**	36.4 ± 3.2*#	<0.001
		Iron deficiency anemia	11.7±0.7*§	6.4 ± 0.5**	4.7 ± 0.7	3.7 ± 0.8	3.6 ± 1.2	<0.001
		Non-iron deficiency anemia	2.9±0.3*	3.7 ± 0.4*	5.8 ± 0.6	10.3 ± 1.3*	32.8 ± 3.0*#	<0.001
	Model 2	Anemia	15.0±0.8*§	10.0 ± 0.7	10.0 ± 1.0	13.2 ± 1.6	35.4 ± 3.2*#	<0.001
		Iron deficiency anemia	10.9±0.8*§	6.6 ± 0.6	5.5 ± 0.8	5.0 ± 1.0	5.5 ± 1.4	<0.001
		Non-iron deficiency anemia	4.2±0.4	3.4 ± 0.4	4.5 ± 0.6	8.2 ± 1.3*	29.9 ± 3.1*#	<0.001
	Model 3	Anemia	15.2±0.8*§	10.0 ± 0.7	9.8 ± 1.0	12.9 ± 1.6	35.1 ± 3.3*#	<0.001
		Iron deficiency anemia	11.0±0.8*§	6.6 ± 0.6	5.4 ± 0.8	5.0 ± 1.0	5.5 ± 1.0	<0.001
		Non-iron deficiency anemia	4.2±0.4	3.5 ± 0.4	4.4 ± 1.6	7.9 ± 1.3**	29.6 ± 3.1*#	<0.001
	Model 4	Anemia	14.8±0.8*§	10.6 ± 0.7	10.0 ± 1.0	12.5 ± 1.6	32.9 ± 3.2*	<0.001
		Iron deficiency anemia	10.8±0.8*§	6.8 ± 0.6	5.5 ± 0.8	4.8 ± 1.0	4.5 ± 1.4	0.004
		Non-iron deficiency anemia	4.0±0.4	3.8±0.4	4.5±0.6	7.7±1.3**	28.3±3.0*	<0.001
	Model 5	Anemia	14.0±0.8**,§§	11.2±0.7	10.5±1.0	13.2±1.6	32.3±3.2*	<0.001
		Iron deficiency anemia	10.2±0.8*,§	7.3±0.6	5.9±0.8	5.5±1.0	4.0±1.5	0.004
		Non-iron deficiency anemia	3.8±0.4	3.9±0.4	4.6±0.6	7.7±1.3**	28.3±3.0*	<0.001

In both sex, the prevalence of anemia in the group of an eGFR>105–89 mL/min/1.73m^2^and <59 mL/min/1.73m^2^ were significantly higher than that in other groups after adjustment of various factors. In men, the prevalence of non-iron deficiency anemia showed similar pattern with that of anemia, but there was no difference in the prevalence of iron deficiency anemia. In women, the prevalence of iron deficiency anemia was highest in the group of an eGFR≥105 mL/min/1.73m^2^. The prevalence of non-iron deficiency anemia was significantly higher in the group less than eGFR 75 mL/min/1.73m^2^than other groups. After adjustment, the hemoglobin level was the highest in the group of an eGFR of 75–89 mL/min/1.73m^2^in both sex. P value means P for difference.

* P<0.01 vs. Group 3

** P<0.05 vs. Group 3

§ P<0.01 vs. Group 2

§§ P<0.05 vs. Group 2

# P<0.01 vs. Group 4.

## Discussion

In this nation-wide representative population-based analysis, the prevalence of anemia was 2.6% (IDA: 0.7%) in men and 12.8% (IDA: 8.0%) in women. The study showed that the estimated hemoglobin level increased with a decrease in the eGFR; this value then peaked at an eGFR of 60–89 mL/min/1.73m^2^ and decreased thereafter, following the adjustment for several factors; the estimated prevalence of anemia at an eGFR of 60–104 mL/min/1.73m^2^ was significantly lower than that at an eGFR of ≥105 mL/min/1.73m^2^; and the weighted prevalence of anemia at an eGFR of <60 mL/min/1.73 m^2^ was markedly higher than that at an eGFR of 60–104 mL/min/1.73m^2^.

Only a few reports have described the association between hemoglobin concentration and a minor decline in eGFR in patients with CKD stage 1 and 2. In the Kidney Early Evaluation Program (KEEP) study, the serum hemoglobin levels were reported to be 13.5, 13.7, and 13.5 g/dL in CKD Stages 1,2, and 3, respectively [1]. NHANES III showed a slight elevation in the hemoglobin level at an eGFR of 60–89 mL/min/1.73m^2^, through a graph of hemoglobin percentile versus eGFR [3]. The Atherosclerosis Risk in Communities (ARIC) study showed a subtle increase in hemoglobin concentration and decrease in anemia prevalence at an eGFR of 75–89 mL/min/1.73m^2^ (hemoglobin concentration of 13.1±1.3 at an eGFR of ≥90, 13.3±1.2 at an eGFR of 75–89, 13.1±1.4 at an eGFR of 60–74, and 12.6±1.4 g/dL at an eGFR of 30–59 mL/min/1.73m^2^; anemia prevalence of 22.1% at an eGFR of ≥90, 16.5% at an eGFR of 75–89, 23.9% at an eGFR of 60–74, and 37.5% at an eGFR of 30–59 mL/min/1.73m^2^)[12]. Recently, in an analysis of 145,865 adults who visited a health promotion center in Korea, Oh et al reported that a higher hemoglobin level was associated with a subtle decline in renal function at an early CKD stage [6]. Their study indicated a peak hemoglobin level at an eGFR of 50–69 mL/min/1.73m^2^ (women: mean [95% CI], 12.91 [12.88–12.96] at an eGFR of ≥100, 12.98 [12.96–13.01] at an eGFR of 90–99, 12.98 [12.99–13.03] at an eGFR of 80–89, 13.16 [13.15–13.18] at an eGFR of 70–79, 13.32 [13.28–13.31] at an eGFR of 60–69, and 13.41 [13.31–13.38] g/dL at an eGFR of 50–59 mL/min/1.73m^2^; men: mean [95% CI], 15.12 [15.09–15.16] at an eGFR of ≥100, 15.25 [15.22–15.27] at an eGFR of 90–99, 15.34 [15.32–15.35] at an eGFR of 80–89, 15.46 [15.45–15.47] at an eGFR of 70–79, 15.59 [15.58–15.61] at an eGFR of 60–69, and 15.62 [15.58–15.65] g/dL at an eGFR of 50–59 mL/min/1.73m^2^) [6]. All these findings were comparable with our results.

However, the above-mentioned studies have certain limitations that preclude the drawing of conclusions regarding this issue. First, the main purpose of the analyses was not the investigation of the association between hemoglobin concentration and minor decline in eGFR in population with eGFR greater than 60 mL/min/1.73m^2^, except for the study of Oh et al. For example, the main aim of the ARIC study was to investigate the association of kidney function and hemoglobin level with left ventricular morphology. Second, the KEEP, ARIC, and Oh et al studies were not representative of the general population. As the KEEP and ARIC studies involved a screening program that targeted the high-risk population, the prevalence of obesity, diabetes, hypertension, and cardiovascular risk factors would be much higher in the samples of these studies than in the general population. Moreover, the study of Oh et al analyzed participants who visited a health promotion center for a general check-up. Third, to examine the changes in hemoglobin level according to the decline of renal function alone, we need to adjust for several variables that could potentially affect the hemoglobin level. The adjustment of several parameters (age, college graduation, smoking history, cancer history, degree of obesity, serum lipid level, blood pressure, and menstruation history of women) in the present study facilitated the observation of a more direct association between the hemoglobin level and mild decline in renal function, with minimal concerns regarding confounding effects. Furthermore, as the prevalence of anemia is markedly higher in women than in men, it would be desirable to perform analyses after stratification according to sex. A major salient feature of this study is that it is the first report regarding the association of hemoglobin concentration and anemia prevalence with a minor decline in eGFR at earlier CKD stages from a nation-wide representative population after stratification according to sex and adjustment for several confounding factors.

The main causes of anemia in advanced CKD may be multifactorial, and include erythropoietin deficiency, uremia-induced inhibition of erythropoiesis, shorter red blood cell survival, and iron metabolism disorders [13–15]. Although the pathogenesis of anemia during early CKD is unclear, angiotensin II is suggested as a possible cause of tissue hypoxia during early CKD. Initial hypoxia in a remnant kidney model is dependent on the activation of the renin-angiotensin system and hemodynamic alterations after nephron loss [16]. The activated renin-angiotensin system induces tubular sodium reabsorption and vasoconstriction of the afferent arteriole, resulting in higher oxygen consumption and relative tubular hypoxia [17–19]. Peritubular cells sense low oxygen tension, produce hypoxia-inducible factor and erythropoietin, thus increasing hemoglobin synthesis [20–22]. However, it is conflicting whether serum erythropoietin level is increased or not in patients with eGFR greater than 60 mL/min/1.73m^2^ [23,24]. In the present study, the prevalence of IDA did not differ according to eGFR in men, and but decreased according to eGFR in women. Therefore, the influence of certain causes of anemia, other than IDA, should be clarified in patients with early CKD.

Anemia is a well-known risk factor for cardiovascular morbidity and all-cause mortality in patients with CKD [25,26]. Anemia is also important in patients with an eGFR of >60 mL/min/1.73m^2^. In particular, among non-CKD patients with heart failure, a hemoglobin level of <13.0 g/dL is an independent risk factor of death [27]. However, patients with a hemoglobin level of <14 g/dL also show a higher mortality rate as compared to those with hemoglobin levels of 14–14.9 or >15 g/dL [28]. These results indicate that survival could be affected by subtle changes in the hemoglobin concentration, or even at a normal hemoglobin level, in patients with CKD stage 1 or 2.

This study has several limitations. The main limitation is that it is performed on a limited cross-section of the population. To clarify the causal relationship between early renal dysfunction and hemoglobin level, a longitudinal prospective study is needed. Second, we used the change in GFR based on serum creatinine level as a marker of renal dysfunction. However, the use of creatinine to estimate GFR has certain limitations in terms of tubular secretion of creatinine and the variability in creatinine generation between individuals and for the same individual [29]. Although serum cystatin C or ^51^Cr-EDTA GFR can be used to validate an eGFR, these tools are not available in our study. Third, the history of specific drug use, which might affect the hemoglobin level or eGFR, was not available. Fourth, no information on iron supplementation was available.

In conclusion, the estimated prevalence of anemia in Korean adults was approximately 2.6% in men and 12.8% in women. The data indicate a compensatory increase in hemoglobin level with a minor decline in kidney function, prior to a marked decrease in hemoglobin level with severe renal dysfunction (eGFR < 60 mL/min/1.73m^2^). However, the lower prevalence of anemia with an eGFR of 60–104 mL/min/1.73m^2^ could not be explained by the difference in IDA prevalence. Nevertheless, larger prospective studies of the association between hemoglobin level and early renal dysfunction, as well as re-analysis of the datasets of previous cohort studies, are required.
